# The effect of topical endometrial scratching on pregnancy outcome in women with previous failure of intrauterine insemination: A non-randomized clinical trial

**DOI:** 10.18502/ijrm.v19i5.9256

**Published:** 2021-06-23

**Authors:** Farahnaz Farzaneh, Farzaneh Khastehfekr

**Affiliations:** ^1^Infectious Disease and Tropical Medicine Research Center, Zahedan University of Medical Sciences, Zahedan, Iran.; ^2^Endometriosis Research Center, Iran University of medical Sciences, Tehran, Iran.; ^3^Zahedan University of Medical Science, Zahedan, Iran.

**Keywords:** Infertility, Endometrial, Scratch, Insemination.

## Abstract

**Background:**

The prevalence of infertility is increasing worldwide and the treatment is one of the important issues.

**Objective:**

This study aimed to evaluate the effect of local endometrial scratching on pregnancy outcomes in women with previous failure of intrauterine insemination.

**Materials and Methods:**

This non-randomized clinical trial study was performed on 336 women referred to the infertility clinic of Ali ebn-e Abitaleb Hospital of Zahedan (between May and November 2019). Women were divided into two groups: endometrial scratch as case and a control group. In the case group (n = 173), endometrial scratching was performed on days 8–9 of the menstrual cycle in addition to routine infertility treatments, while in the control group (n = 163), only routine treatment was performed. Chi-square test was used to compare the frequency of male factor severity and the percentage of successful pregnancies between both groups and was used to investigate the effect of male factor on the fertility rate in each group (moderate male factor and mild male factor).

**Results:**

The mean age of the women was 28.4 ± 5.2 yr. The success rate of pregnancy in the case group was 12.3% and in the control group 11%, which were not statistically significant (p = 0.697).

**Conclusion:**

Overall, the results of this study showed that endometrial scratching had no effect on the pregnancy rate.

## 1. Introduction

Infertility is defined as a failure to conceive after one year of regular unprotected intercourse. The incidence of infertility has been reported in different studies ranging from 10% to 15% and can be caused due to male factor, female factor or a mix of both, or in some cases due to an unexplained cause. Moreover, among the causes of infertility due to female factor, ovulation disorders are the most common cause (80%) (1–3).

IUI is the procedure in which prepared semen are entered directly into a woman's uterus, with or without ovarian stimulation.

Owing to the relatively high prevalence of infertility and the high cost of infertility treatments, finding ways to improve infertility treatment is important. Creating a localized injury to the uterine endometrium is one of the methods that can be used to increase the success rate of artificial insemination procedures. The idea originates from some laboratory studies on animals (3).

Endometrial injury and scratching is one such procedure that has been investigated in recent years as a method to increase the implantation rate. Several theories have been put forward for this purpose. The first theory is that the scratching mechanism induces endometrial decidualization, which is able to improve implantation of embryos; the second theory is that the repair process due to endometrial injury involves an inflammatory response mediated by cytokines, growth factors, interleukins, macrophages, and dendritic cells that contribute to embryo implantation. While a significant effect of endometrial scratching has been reported in some of these studies (4, 5), in others, it has been reported to have no effect on pregnancy rate (6, 7). The aim of this study was to investigate the effect of local endometrial scratching on pregnancy outcome in women with previous failure of intrauterine insemination (IUI).

## 2. Materials and Methods

In this non-randomized clinical trial study, 336 infertile women (173 in the case group and 163 in the control group), referred to the infertility clinic of Ali ebn-e Abitaleb Hospital of Zahedan between May and November 2019 were enrolled.

The inclusion criteria of the study were: women aged 18–40 yr, BMI < 30 kg/m${}^{2}$, and 1 < AMH < 3.5 with normal thyroid and prolactin functional tests who were candidates for ovulation induction and IUI and had failed once or twice in these methods despite proper ovulation and primary and secondary infertility with having at least one patent fallopian tube, normal semen analysis, and mild oligospermia. Exclusion criteria, on the other hand, included women who refused to give consent to participate in the study, poor-response women (AMH < 1 ng/dl), with thyroid dysfunction, high serum prolactin, and double blocked fallopian tubes or hydrosalpinx, and those with polycystic ovarian disease (PCOD).

All women who had experienced two or more IUI failures and were eligible for the study were consulted to participate. On days 3–7 of the menstrual cycle, after performing the ultrasound (Philips) by the infertility fellowship, 2 letrozole 2.5 mg or clomiphene 50 mg (Iran Hormone Pharmaceutical Co.) daily was prescribed for five days. Gonadotropin HMG or Gonal-f 75 IU (Karma) was administered 3-4 daily from 7-10 cycles per day according to the women's needs; next, 5–7 days after the last dose of letrozole or clomiphene, a transvaginal ultrasound was performed to evaluate the follicle size, endometrial thickness, and pattern. When the follicles reached a size of 18 mm, HCG (Karma) was injected intramuscularly and 36–48 hr later, a semen sample prepared through gradient process with a volume of 0.5–0.7 cc was injected into the uterus using an IUI catheter. Fourteen days after the insemination, the biochemical pregnancy rate was measured with serum beta HCG.

In the treated group (women in odd days), endometrial scratching was performed on days 8–9 of menstrual cycle using Pipelle catheter (Tasnim Behbod Arman Company), while the control group (women in even days) received routine infertility treatment for IUI without any intervention; randomizing of the two groups was not performed.

### Ethical considerations

The current study was approved by the Ethics Committee of the Zahedan University of Medical Sciences (code: IR. ZAUMS.REC.1397.144) and has been registered in the Iranian Registry of Clinical Trials (Code: IRCT20180731040659N1). In addition, a written informed consent was obtained from all participants.

### Statistical analysis

Data were entered into the Statistical Package for the Social Sciences, version 21.0, SPSS Inc., Chicago, Illinois, USA (SPSS) and analyzed by Chi-square test. P-value < 0.05 was considered as statistically significant.

## 3. Results

**Figure 1:**
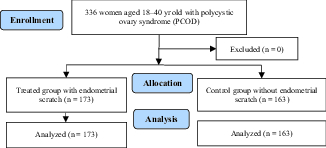
The CONSORT flowchart of the study.

In this study, 336 infertile women referred to the infertility clinic of Ali ebn-e Abitaleb Hospital were studied. The mean age of the patients was 28.4 ± 2.5 yr; of the 336 women studied, 173 were included in the case group and 163 in the control group (Figure 1). The mean duration of infertility was 5.2 ± 3.1 yr, out of the 336 studied patients, 65.5% had mild and 34.5% had moderate male factor. Chi-square test was used to compare the frequency of male factor severity in both groups. A significant difference was observed in the male-factor severity between groups (p = 0.028%, Table I). In this study, the pregnancy rate was reported as 12.3% and 11% in the case and control group, respectively.

Moreover, Chi-square test was used to compare the percentage of successful pregnancies between the two groups, which was not significantly different, the percentages being 11% (19) in the treated group and 12.3% (20) in the control group (p = 0.697).

Since there was a significant difference between the treated and control groups with respect to male-factor severity, Chi-square test was used to investigate the effect of male factor on the fertility rate in each group (moderate male factor and mild male factor). It showed that male factor intensity had no effect on fertility outcome (Table II).

**Table 1:** Comparison of male factor severity in treated and control groups


[1.455in,lr]**Group** **Male-factor severity**	**Mild**	**Moderate**	**P-value**
**Control**	114 (70%)	49 (30%)	0.028
**Treated**	106 (62%)	67 (38%)	
Chi-square Test			

**Table 2:** Comparison of pregnancy success rates between the two study groups in patients with mild and moderate male factor


**Pregnancy, studied group**		**Positive**	**Negative**	**P-value**
**Mild oligospermia**				
	**Control**	11 (9.6)	103 (90.4)	0.686
	**Treated**	12 (11.3)	94 (88.7)	
**Moderate oligospermia**				
	**Control**	7 (16.7)	35 (83.3)	0.361
	**Treated**	7 (10.6)	59 (89.4)	
Data presented as n (%). Chi-square test				

## 4. Discussion

The aim of this study was to evaluate the effect of local endometrial scratching on the pregnancy outcome of 336 infertile women (173 in the treated group and 163 in the control group) referred to infertility clinic of Ali ebn-e Abitaleb Hospital of Zahedan with a previous failure of IUI. The mean age of the women was 28.4 ± 2.5 yr. About 65.5% of cases had mild and 32.1% had moderate male factor. The pregnancy success rate was 12.3% in the treated group and 11% in the control group. According to the Chi-square test, there was no significant difference between the two groups in terms of fertility.

Barash and colleagues in their study on 134 infertile women in the United States showed that the implantation rate, clinical pregnancy, and live birth rate were significantly higher in the endometrial scratch group than the control group (more than twice) and hence their results are not consistent with our study (8). Similarly, a study conducted by Kara and colleagues in Turkey evaluated the efficacy of local endometrial injury on 41 women, they reported that both the implantation rate and the clinical pregnancy rate in the endometrial scratch group were significantly higher than the control (9). Another retrospective study conducted by Dain and colleagues in a multicenter cohort in Ukraine, London, and Israel on 737 oocyte donation cycles reported that there was no significant difference in the live birth rate and clinical pregnancy rate between women with local endometrial injury and the control group (who did not receive endometrial injury), which is similar to our study (10).

Furthermore, in a study conducted by Ashrafi and colleagues in Tehran, which examined the effect of endometrial scratching on fertility outcomes in 150 women with a previous ICSI history, there was no significant difference between the two groups in terms of pregnancy rate. Also, the rate of clinical pregnancy and abortion was not significantly different between the two groups (11).

Contrastingly, a study conducted by Yu Huang and colleagues in China reported that 30 women undergoing endometrial scraping had a pregnancy rate of 100% and only 46% of women without endometrial scratch were pregnant (12).

In another study conducted by Bakshi and colleagues, which examined the effect of endometrial scratch on the outcome of in vitro fertilization cycles in 336 women, reported that endometrial scratch in the first cycle of IVF had significant consequences, but for recurrent cases, this was not a significant consequence (13).

However, some studies have shown that endometrial scratch success in primary and secondary infertility is also not significant (12, 13).

According to the results of the Karimzadeh and colleague's study, local injury to the endometrium on the day of oocyte retrieval disrupts the receptive endometrium and has a negative impact on implantation and IVF outcomes (14), which is consistent with this study.

## 5. Conclusion

Overall, the results of this study showed that endometrial scratching had no effect on the fertility rate. Since the results of the studies in this case are contradictory, it is recommended that further studies be conducted with larger sample size.

##  Conflict of Interest 

The authors declare no conflict of interest regarding the publication of this paper
