# Corrigendum to “The effect of topical endometrial scratching on pregnancy outcome in women with previous failure of intrauterine insemination: A non-randomized clinical trial” [Int J Reprod BioMed 2021; 19: 465-470]

**DOI:** 10.18502/ijrm.v22i3.16169

**Published:** 2024-05-15

**Authors:** Farahnaz Farzaneh, Farzaneh Khastehfekr

The publisher has been informed of an error that occurred on page 465 in which the first author affiliation order is incorrect and should be replaced with each other. On behalf of the author, the publisher wishes to apologize for this error. The online version of article has been updated on 31 March 2024 and can be found at https://doi.org/10.18502/ijrm.v19i5.9256.

